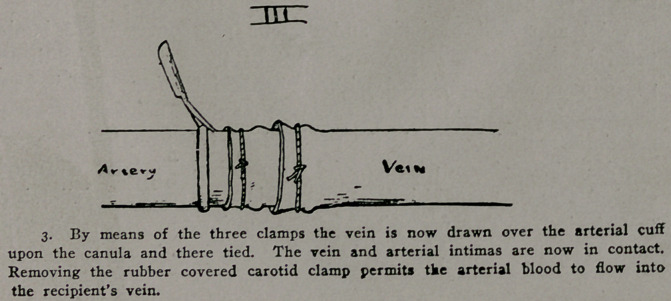# The Transfusion of Blood

**Published:** 1911-02

**Authors:** H. P. Cole

**Affiliations:** 202-204 Conti St., Mobile, Ala.


### Journal-Record of Medicine

Successor to Atlanta Medical and Surgical Journal, Established 1855, and Southern Medical Record, Established 1870.

OWNED BY THE ATLANTA MEDICAL JOURNAL CO.

Published Monthly

*Official Organ Fulton County Medical Society, State Examining Board, Presbyterian Hospital, Atlanta, Birmingham and Atlantic Railroad Surgeons' Association, Chattahoochee Valley Medical and Surgical Association, Etc.*

EDGAR G. BALLENGER., M. D., Editor.
BERNARD WOLFF, M. D., Supervising Editor.
A. W. STIRLING, M. D., C. M., D. P. H., J. S. HURT, B. Ph., M. D.
GEO. M. NILES, M. D., W. J. LOVE, M. D., (Ala.); Associate Editors.
E. W. ALLEN, Business Manager.

COLLABORATORS

Dr. W. F. WESTMORLAND, General Surgery.
F. W. McRAE, M. D., Abdominal Surgery.
H. F. HARRIS, M. D., Pathology and Bacteriology.
E. B. BLOCK, M. D., Diseases of the Nervous System.
MICHAEL HOKE, M. D., Orthopedic Surgery.
CYRUS W. STRICKLER, M. D., Legal Medicine and Medical Legislation.
E. C. DAVIS, A. B., M. D., Obstetrics.
E. G. JONES, A. B., M. D., Gynecology.
R. T. DORSEY, Jr., B. S. M. D., Medicine.
L. M. GAINES, A. B., M. D., Internal Medicine.
J. N. LeCONTE, M. D., Disease of the Stomach and Intestines.
L. B. CLARKE, M. D., Pediatrics.
EDGAR PAULIN, M. D., Opsonic Medicine.
THEODORE TOEPEL, M. D., Mechano Therapy.
R. R. DALY, M. D., Medical Society.
A. W. STIRLING, M. D., etc., Diseases of the Eye, Ear, Nose and Throat.
BERNARD WOLFF, M. D., Diseases of the Skin.
E. G. BALLENGER, M. D., Diseases of the Genito-Urinary Organs.

VOL. LVI. FEBRUARY, 1911 NO. 11.

### THE TRANSFUSION OF BLOOD. REPORT OF TWENTY CASES.*

BY H. P. COLE, M. D., MOBILE, ALA.

Transfusion of blood in the treatment of disease is not a new procedure. Herophilus in his treatise on anatomy made a definite reference to their operation. Pope Innocent VIII was transfused with the blood of three youths in April, 1492, in an unsuccessful attempt to prolong his life. As early as 1615 Labavius gave an accurate description of blood transfusion.

These facts present an interesting commentary upon the universal custom of accrediting Harvey with the discovery of

*Read before the Chattahoochee Valley Medical and Surgical Association at Columbus, Georgia, Jan. 12, 1911.

blood circulation in 1616. It is inconceivable that these early investigators could have devised a method of transfusion or conceived any indication for its employment without a very definite knowledge both of the circulatory system and the physiological properties of blood.

It is only within recent years that the suture method of Carrell and the canula method of Crile have made feasible the wide application that transfusion enjoys to-day in the treatment of a number of surgical conditions.

Transfusion has met its greatest success perhaps in the treatment of acute hemorrhage, enabling us to convert many unfavorable surgical risks into favorable ones. Various types of secondary anemias, infantile hemorrhage and surgical shock are a few of the conditions in which transfusion has met with success.

In an effort to determine the value of transfusion in severe cases of pellagra, I employed the procedure in a terminal case on August 4th, 1908. The recovery in this case was of such decided nature that we have since transfused 19 additional cases. Dr. Gilman J. Winthrop of Mobile, Ala., has collaborated in this work.

We have endeavored to preserve a spirit of unbiased judgment throughout our investigations, and we present the following data to the profession, for the benefit of those wishing to draw conclusions from our cases.

All of these cases have been transfused only in the last stages of pellagra. To illustrate the type of cases in which transfusion has been employed, it is sufficient to state that two cases were moribund at the time of operation; one died on the railway train 20 minutes before arriving at Mobile for transfusion.

| | | |
|---|---|---|
| Cases transfused ........... | 20, | Ages 3 to 53. |
| Recoveries ................ | 12, | 60% |
| Deaths .................... | 8, | 40% |

Among the eight deaths there was improvement after transfusion in only two cases. These two cases presented complications incompatible with recovery; one complicated by tubercular peritonitis showed temporary improvement for several days, but died one month after operation. The second case, a boy, aged three, showed distinct improvement following transfusion, but died eight days later from intestinal perforation. Two of the fatal cases received an inadequate amount of blood at transfusion, due to the unsuitable donors available.

When we consider the grave mortality rate (80 to 90%) in the terminal cases of pellagra which are treated by medical measures, we cannot but feel that our mortality rate of 40%, by transfusion offers a far greater hope of recovery in the grave type of cases.

We have never observed hemolysis, agglutination, thrombosis nor embolism in any of the cases as a result of transfusion.

All of the cases presented distinct severe lesions of pellagra; there was marked emaciation, asthenia, anemia, reflex excitability and in many cases grave mental changes. Severe pellagra erythema, stomatitis and diarrhea were present in a majority of cases.

The most suggestive evidence of the value of transfusion lies in the fact that there was alleviation or disappearance of all of the symptoms of pellagra in the 14 cases that recovered, within a few days or weeks following transfusion. In several cases there was an astonishing improvement in the patient's mental condition shortly following operation. In all of the recovered cases there was a rapid increase in hemoglobin index, rapid return to body strength, return of digestive faculties, and increase of body weight. These cases gained from 3 to 8 1-2 pounds in the first week following transfusion, one case gaining 34 pounds in 11 weeks. Only one of the 14 cases has relapsed. This patient relapsed two years after making a complete recovery and remained free from symptoms for a period of nearly two years. As this patient was constantly exposed to pellagra, it is fair to assume that this case suffered a second attack rather than a relapse.

All approved hygienic and dietetic procedures were instituted in the post-operative treatment of these cases. As these procedures were employed without beneficial effect previous to transfusion, it is plausible to assume that these procedures were not the essential factors in the recoveries, after transfusion.

We have been unsuccessful in ascertaining any constant clinical sign that would indicate a certainty of recovery following transfusion. The necessity of transfusion can only be ascertained upon the appearance of positive retrogression, after careful obervation and the employment of all approved institutional and constitutional therapeutic agents.

A majority of the cases referred us for transfusion have recovered through hygienic and supportive treatment, without resorting to transfusion.

The donors selected to furnish blood for these cases have, in the majority of cases, lived in the same environment as the patients. We have selected this type of donor with the assumption that such donors possess a certain degree of immunity. In a few cases we have been able to employ donors who had recovered from pellagra. We have observed no greater per cent. of recovery when transfusing from a donor who has had pellagra. We have noted no necessity of employing a relative as a donor. Non-relatives as donors have given as great a percentage of recovery.

The operation is performed under cocaine anaesthesia. The transfusion (controlled by a soft clamp), continues from 20 to 40 minutes, depending upon the cardiac condition of the recipient and donor. We have occasionally bled several ounces of venous blood from the recipient, before transfusion. Syncope should be guarded against in the donor and acute dilatation of the heart watched for in the recipient. The Trendelenberg posture is indicated for syncope, and the reversed Trendelenberg for acute dilatation. We have never observed either condition.

### Conclusion.

After transfusing 20 cases of pellagra in the terminal stages and noting no complications resulting from the operations, we feel we may safely resort to transfusion in the terminal stages of the disease when all other approved therapeutic agents have been employed without avail.

There is no necessity of selecting a relative as donor or selecting a donor who has recovered from pellagra.

We are unable to say that there is any immune principle transfered by transfusion. The rapid alleviation of symptoms, increase in hemoglobin index and increase of body weight indicate that the beneficial results are attributable to the relief of the existing anemia.

The transfusion must be undertaken with a full knowledge of the difficulty and danger of the operation, and careful post-operative supportive treatment must be instituted for a considerable period of time following the transfusion.

202-204 Conti St., Mobile, Ala.

## Figures and Tables

**I f1:**
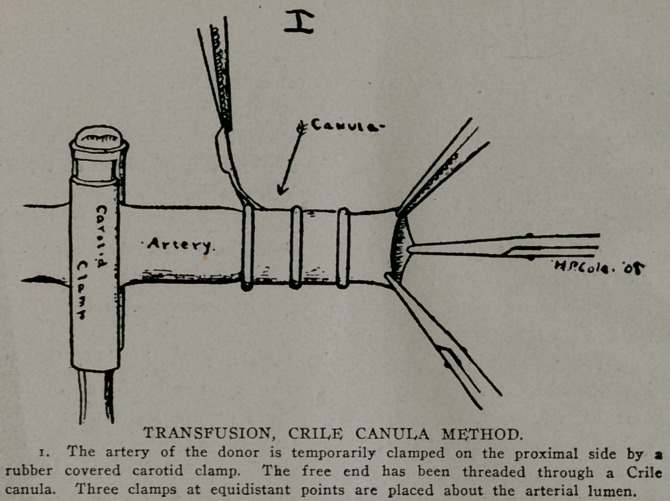


**II f2:**
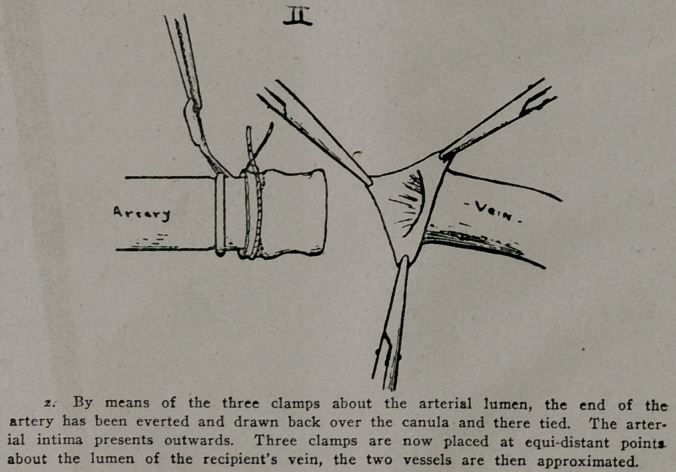


**III f3:**